# Raman spectroscopy as a tool for ecology and evolution

**DOI:** 10.1098/rsif.2017.0174

**Published:** 2017-06-07

**Authors:** Arno Germond, Vipin Kumar, Taro Ichimura, Jerome Moreau, Chikara Furusawa, Hideaki Fujita, Tomonobu M. Watanabe

**Affiliations:** 1 RIKEN Quantitative Biology Center, 6-2-3 Furuedai, Suita, Osaka 565-0874, Japan; 2 Université de Bourgogne Franche Comté, UMR CNRS 6656 Biogeosciences, Equipe Ecologie Evolutive, 6 Boulevard Gabriel, Dijon 21000, France; 3 Universal Biology Institute, The University of Tokyo, 7-3-1 Hongo, Tokyo 113-0033, Japan; 4 WPI Immunology Frontier Research Center, Osaka University, 1-3 Yamadaoka, Suita, Osaka 565-0871, Japan

**Keywords:** Raman spectroscopy, vibrational imaging, experimental evolution, ecology, phenotyping, pigment

## Abstract

Scientists are always on the lookout for new modalities of information which could reveal new biological features that are useful for deciphering the complexity of biological systems. Here, we introduce Raman spectroscopy as a prime candidate for ecology and evolution. To encourage the integration of this microscopy technique in the field of ecology and evolution, it is crucial to discuss first how Raman spectroscopy fits within the conceptual, technical and pragmatic considerations of ecology and evolution. In this paper, we show that the spectral information holds reliable indicators of intra- and interspecies variations, which can be related to the environment, selective pressures and fitness. Moreover, we show how the technical and pragmatic aspects of this modality (non-destructive, non-labelling, speed, relative low cost, etc.) enable it to be combined with more conventional methodologies. With this paper, we hope to open new avenues of research and extend the scope of available methodologies used in ecology and evolution.

## 1. Introduction

Ecology and evolution are interlinked disciplines and aim together to decipher the complexity of biological systems at various spatial and temporal scales. Fundamentally, ecology aims at characterizing the relation between biological systems (cell, individual, community, population, etc.) and their environment as shaped by selective pressures. To complement this effort, experimental evolution tests how the selective pressures identified from ecological studies drive the evolution of these biological systems. Contributing to this interplay is the introduction of novel techniques granting us new vantage points which offer new perspectives on biological systems.

In this perspective, we explore the possibilities offered by Raman spectroscopy in the context of ecology and experimental evolution and argue that this optical modality can benefit researchers of both communities. Vibrational spectroscopy techniques such as Raman spectroscopy provide a unique molecular signature of a sample (inorganic or organic) in a label-free, non-destructive and reproducible manner [1]. Recently, Raman spectroscopy has been increasingly reported in biological and biomedical studies for the purposes of identification, classification, diagnosis and microscopic imaging of living or fixed single cells, tissues and organisms [2–5]. As Raman spectroscopy is recognized as a reliable technique for characterizing species and biological processes, we envision that Raman spectroscopy can provide relevant molecular information in the context of ecology and experimental evolution, thus feeding the dynamic interplay existing between these fields.

Surprisingly, Raman spectroscopy has not yet been widely introduced in ecology and experimental evolution, mainly because of a lack of intersecting connections between these fields and the Raman community. Rare exceptions are however emerging, as described later in the text, and we believe it is time to generalize this technique to ecologists and evolutionists, who have much to gain from the integration of this optical modality. In doing so, we place Raman spectroscopy at the heart of the conceptual, technical and pragmatic considerations of ecology and experimental evolution.

## 2. A brief overview of Raman spectroscopy

**Figure 1.**
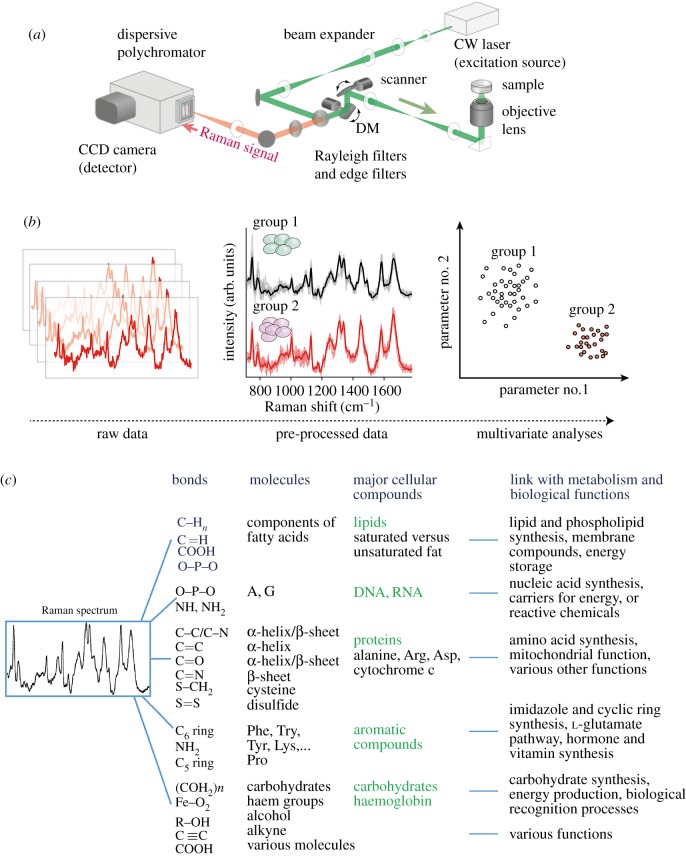
A general overview of Raman spectroscopy. (*a*) A generalized overview of the optical set-up for a typical spontaneous Raman spectroscopic microscope. A monochromatic laser light illuminates the sample, emitting Raman scattering light. A small portion of this scattering passes through the objective lens and goes through the optical pathway to the dispersive polychromator (i.e. spectrophotometer), where it is captured by a CCD detector. (*b*) Raw spectral data must be processed through various procedures to improve the quality of the signal by removing noise (e.g. due to cosmic rays, the auto-fluorescence signal of the samples or from unavoidable technical variations). Because spectra are not always useful to visualize and are characterized by the molecular composition between each group or conditions, they are usually used as the input for multi-variate analyses such as principal component analyses, projection latent structure analyses, discriminant analyses and support vector machines, which are particularly powerful for identifying and discriminating individuals from the contribution of all or specific Raman wavelengths. (*c*) Representation of the inherent layers that contribute to the complex spectral signature.

Raman spectroscopy is a vibrational spectroscopy technique used to collect a unique chemical fingerprint of molecules from living or non-living samples (figure 1). In the case of an optical Raman microscope, a laser is used as an excitation source and is targeted on a biological sample, causing the emission of Raman scattering. A fraction of this scattering signal is captured back through the objective lens and measured by a polychromator equipped with a multi-channel detector (figure 1*a*). Each molecule has a distinct set of vibrational energy levels, and their individual contribution yields a spectral signature within a few seconds of exposure. From the Raman spectrum of biological samples, the major cellular compounds (DNA/RNA, proteins, lipids, carbohydrates, chlorophylls, carotenoids, etc.) can be identified due to their wavelength-specific position. While the information from individual peaks (or their ratio) provides useful information, multi-variate analyses on whole spectra are often used to discriminate physiological differences among individuals or groups (figure 1*b*). Although the compound effect of the inherent layers constituting the Raman signal remains to be characterized (figure 1*c*), the interplay between these layers contributes to a rich and complex spectral signature that monitors the changes in the molecular composition and metabolism of living organisms or cells. Prominently, Raman spectroscopy has demonstrated its ability to identify microorganisms, algae, fungi and whole organisms and also to characterize biological phenomena in a reproducible and very accurate manner [6–9].

The low intensity of Raman scattering signals, inherently weaker than fluorescence signals, has historically limited the application of Raman spectroscopy to the identification of geological and synthetic compounds [10]. However, technical advances in terms of detection limits (i.e. spectral and spatial resolution, sensitivity, speed of acquisition) have strongly contributed to improving the sensitivity of the method and allow biological applications. In particular, the use of a coupled charge device (CCD) camera or an sCMOS camera with high quantum yield and low dark current, and an objective lens with a high optical aperture (N.A.), has greatly contributed in reducing the exposure time/laser intensity required to obtain a spectrum with a good signal-to-noise ratio. As for the spatial resolution of a confocal Raman microscope, it is determined in the same manner as a confocal fluorescence microscope and depends on the N.A. of the objective and the excitation laser wavelength. With an excitation wavelength of 532 nm, a 1.2 N.A. objective lens and a slit size set around the focal spot size, the lateral (*XY*) spatial resolution is approximately 270 nm, which enables subcellular compartments of living single cells and tissues to be imaged (e.g. [4]).

It is worth mentioning that, following these technical improvements, a multitude of derivatives of vibrational Raman scattering have been developed, such as coherent anti-Stokes Raman scattering, surface-enhanced Raman scattering, tip-enhanced Raman scattering and stimulated Raman scattering, each providing different experimental advantages and limitations that must be considered for the biological entity of interest [2]. Raman spectroscopy has also been criticized for the interpretability of its spectral information. For example, it is currently not possible to identify precisely the type or concentration of a given protein present in a sample, although other compounds such as pigments may exhibit very specific spectral signatures. Efforts have been made, however, to facilitate the processing and interpretation of spectral information, and these efforts are supported by solid theoretical foundations (signal processing, multi-variate analyses, etc.).

In our opinion, these recent technical and analytical developments have only started to reveal the full possibilities of interpreting biological phenomena from Raman spectral data, as demonstrated by the ever-growing list of applications and discoveries in various fields. We wish to further this effort in ecology and evolution.

## 3. Grounding Raman spectroscopy in the conceptual framework of ecology and experimental evolution

The choice of a given technique in ecology is fundamentally constrained by its capacity to characterize specific traits and distinguish variations among the individuals or samples under consideration, which may differ in response to environmental changes. Thus, the methods used in ecology must be replicable, sensitive and robust. Interestingly, the ability of Raman spectroscopy to identify subtle variations among individuals or samples (species, subspecies, growth state, type of pigment, etc.) for the purpose of discrimination and classification has been widely reported, notably in microbiological studies (e.g. [6,7]). This ability to delineate a fine granularity of phenotypes can also be applied to identify the environmental stressors to which the measured organisms are subjected. For example, specific spectral information from single bacteria cells subjected to antibiotic stressors has been used to establish a barcode reflecting the severity (concentration dependence) and temporality (time dependence) of the responses [8,9]. It is worth mentioning that recent studies showed supporting evidence for the sensitivity of infrared spectroscopy in monitoring *in situ* environmental stress from the tissue samples of various species [11–13]. Likewise, it is expected that the ability of Raman spectroscopy to provide a broad and fine characterization of biological systems will benefit a broad range of ecological studies in both temporal and spatial contexts.

Surely, this aspect is also of importance in the context of experimental evolution. However, it can be argued that the keystone for enabling Raman spectroscopy to be integrated into this field is that the molecular signature must reasonably be linked to fitness or a relevant adaptive response. Interestingly, some studies have hinted at this possibility. For example, spectral taxonomic markers were found to relate to the microbial growth [7], the viability of the cell [14] and changes in the mitochondrial membrane potential associated with apoptosis [15]. As a case in point, a laboratory experiment showed that the somatic growth and reproductive output of a copepod species were directly correlated with its total fatty acid content and with its composition in saturated fatty acids [16]. As Raman spectroscopy is particularly good at detecting lipids, with the possibility of distinguishing saturated lipids from unsaturated ones [17], the spectral information related to lipid markers could be used here as a proxy to estimate the copepod's fitness.

## 4. Implementing Raman spectroscopy in the context of ecology and evolution

In addition to the aforementioned conceptual considerations, it can be argued that experimental design in ecology and evolution takes into account several technical or pragmatic aspects. In this section, we argue that Raman spectroscopy affords the opportunity to counter a number of these limitations.

**Figure 2.**
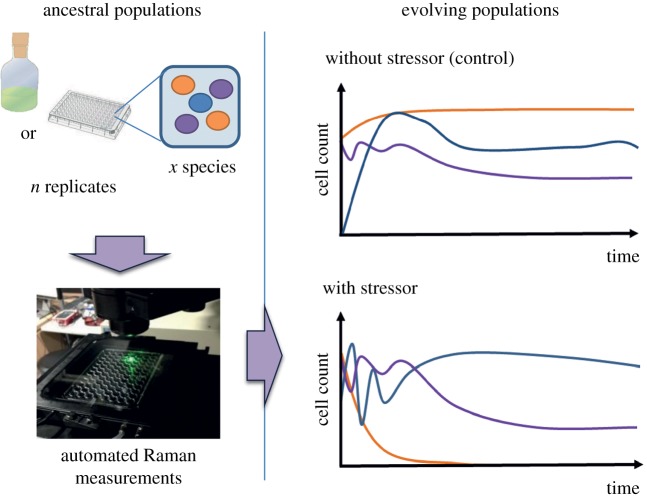
Perspective of microbial experimental evolution using Raman spectroscopy. Long-term cultures composed of single or multiple species are evolved in the presence or absence of varying levels of stressors. Population dynamics monitoring, as well as the characterization and isolation of emerging phenotypes (e.g. antibiotic-resistant mutants), could be performed by Raman spectral analysis, given the condition they exhibit different spectral markers (a specific pigment, amino acids, etc.), as suggested by recent studies [7–9].

Ecological studies often require the collection of information from many individuals to capture general trends while minimizing the interference and confounding effects. For example, non-invasive practices may be required due to ethical considerations for ecological studies (well-being of the studied organisms, endangered species, etc.). By comparison, in experimental evolution, analytical approaches for phenotyping often rely on destructive methods, which increase the need for sampling, labour, costs and the risk of perturbing or contaminating the system (e.g. in microbial cultures) [18]. Thus, non-invasive Raman spectroscopy techniques should be particularly advantageous for the monitoring of populations in laboratory set-ups or even in the field. In particular, we envision that non-invasive Raman spectroscopy techniques should integrate well with the studies of culturable/non-culturable microorganisms. Measurements take only a few seconds for each spectrum, can be fully automatized and can be scaled to various containers (slide, 96-well plate, agar plate, cuvette, etc.), thus minimizing the labour required and the risk of contamination through repeated sampling. For example, this could be particularly useful for characterizing the ecological or evolutionary dynamics of microbial species in long-term experimental ecosystems (e.g. [19,20]). In this context, Raman spectroscopy could be used to monitor the population dynamics and quantify the severity of various selective pressures (figure 2).

One crucial advantage of this non-invasive aspect of Raman spectroscopy is that the sample remains viable for further analytical procedures. Thus, these approaches can be used as an adjunct to more conventional methodologies. This point is particularly relevant to ecology and experimental evolution, which often rely on a combination of phenotypic and genetic analyses at different time points. For example, populations or single cells of microorganisms could be characterized by Raman-activated cell sorters [21], and immediately retrieved for genetic analyses. The range of possible combinations with conventional methods is apparently unlimited. One of the most significant examples is the integration of Raman spectroscopy with fluorescent *in situ* hybridization (Raman–FISH) to reveal further biologically relevant information [22]. This method has been used as a culture-independent approach to determine and quantify the activity of aerobic naphthalene-degrading groundwater microbial communities [23]. By taking advantage of phylogenetic analyses of functional RNAs coupled with Raman imaging to confirm the presence and quantify the activity *in situ* of the degrader pools, this study illustrated the role played by unculturable microorganisms in the biodegradation and the carbon cycle process in the ecosystem. Thus, Raman–FISH has the potential to enable the measurement of genes that affect ecological success, and possibly evolutionary fitness, in natural environments and populations.

**Figure 3.**
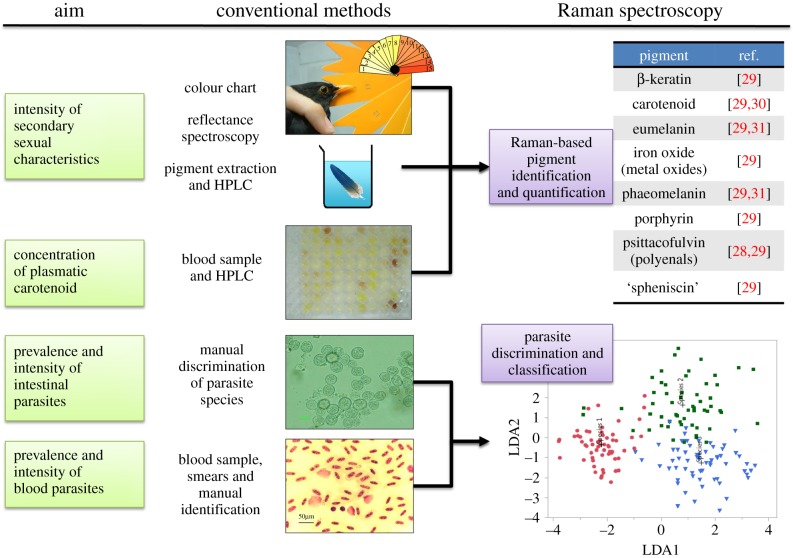
Example of the advantages of Raman spectroscopy in evaluating the effects of food availability and parasitism on the secondary sexual characteristics in birds. In black birds, carotenoid pigments are an indicator of good health as their concentration is directly correlated with food availability and the immuno-resistance against parasitism [32]. Raman spectroscopy is known to provide specific signatures for pigment identification and quantification, as shown in the pioneering work of Thomas *et al*. [29,30], Galvan & Jorge [31] and Fernandes *et al*. [28], thus giving an alternative to the destructive and time-consuming conventional methods currently used by ecologists. This may become useful to monitor indirectly the health status of birds in natural populations. Likewise, the discrimination of intestinal and blood parasite species (copepoda, protozoa, etc.) could be done by spectral measurements in a systematic manner without the need for smears and hazardous manual identification. We show here a hypothetical example of linear discriminant analysis (LDA), in which three groups are identified (each point of the plot corresponds to a spectrum taken from one individual).

The above advantages of Raman spectroscopy are complemented by the diversity of organisms on which it can be used. Specifically, previous studies have successfully applied Raman spectroscopy to the characterization of living or fixed bacteria, mycobacteria, microalgae, fungi, lichen, spores, worms (e.g. *Caenorhabditis elegans*), protists, corals, insects, mice, and also plant tissues (leaves, roots, seeds, etc.) (e.g. [24–29]). Although the totality of the Raman spectra can hold much information to identify intra- or interspecific variations, some studies focus specifically on the identification of specific chemical structures, such as pigments (carotenoids, melanin, chlorophylls, etc.), the unique spectral signatures of which [28–31] can reflect the physiological condition of an individual. For example, Brat *et al*. [26] took advantage of Raman spectroscopy to study the carotenoid composition of living aphid insects raised under various environmental conditions. The carotenoid content correlated with the ATP content, which was used as a proxy marker for the insect's metabolism. Moreover, the authors could demonstrate that environmental stresses conducted on a given population resulted in the phenotypic heterogeneity of carotenoid content two generations later. This kind of use of Raman spectroscopy is particularly relevant as pigments are difficult to extract and quantify by conventional methods. Because of the ubiquity and ecological importance of pigments in insects, phytoplankton, birds, crustaceans, protists, etc., we envision that Raman spectroscopy will open a broad range of applications to address pertinent questions on ecological communities. We show a possible application related to the ecology of birds in figure 3.

Furthermore, rather than focusing on the biological entity, one may instead measure the molecular composition of the environmental niches. For example, Raman spectroscopy has been successfully used to characterize the molecular composition of soil [33]. Thus, we expect Raman spectroscopy will allow assessment of the impact of environmental composition on the biodiversity or functions of resident species. One of the first ecological studies using Raman spectroscopy provides a good example of how the molecular composition of lichen substrata is affected by environmental variables such as salinity, freshwater or rock type, which in turn affect the distribution of lichen species across natural sites [24].

And last but not least, we stress that pragmatism should always be used when choosing an experimental method. Portable Raman spectroscopes are now commercialized, with prices ranging from US$3000 to US$30 000. This could be a strong incentive for ecologists working in the field. However, depending on the molecular compounds or organisms that are being studied, a home-made or commercialized microscope with more expensive equipment might be required to achieve a good spectral resolution and sensitivity (approx. from US$50 000 to US$200 000, depending on the optical set-up). Specifically, for the most demanding biological samples, a good polychromator equipped with a powerful detector (usually, a CCD camera) and a high N.A. objective lens are required. On the other hand, one advantage by comparison with other methods (e.g. figure 3) is that there is little or no need for sample preparation (e.g. extraction, labelling, etc.), which makes the cost of analysis particularly low and appealing. To facilitate the practical use of Raman spectroscopy by biologists, guidelines presenting how to build and set-up Raman microscopes, and how to perform spectral analyses, were recently published (e.g. [1,34]). Another strong incentive for the integration of Raman platforms in academic environments is that they can benefit researchers from various specialties, including cell biology, ecology, chemistry, geology or engineering.

## 5. Perspectives and conclusion

Our work was motivated by the fact that the advantages of Raman spectroscopy have not yet been introduced in ecology and experimental evolution, despite its ability to address many central questions in these fields. Here, we demonstrated that the unique metabolic signature of the Raman signal and its ability to characterize the phenotypes of living and non-living entities show promise for various applications in ecology and experimental evolution. It is worth mentioning that similar conclusions could be drawn with other derivatives of infrared or near-infrared absorption spectroscopy techniques. The non-destructive nature of these techniques, their versatility and their relative low cost make them appealing to a broad range of users and experimental set-ups.

In the future, it is worth exploring whether the aforementioned advantages of vibrational spectroscopy could benefit the empirical demonstrations of current and emerging concepts in ecology and evolution, such as the study of the phenotypic plasticity of individuals and its consequences for evolution [35–37] and for evolutionary rescue [38,39]. The ability of Raman spectroscopy to measure both at various scales (from molecules to populations) may give the opportunity to study complex hierarchical dynamics [40], or even the characterization of the molecular evolutionary dynamics in artificial systems [41].

In order to open new avenues of research and to extend the scope of available methodologies used in ecology and evolution, we strongly encourage ecologists and experimental evolutionists to communicate with spectroscopy experts and discuss the possibility for biologically relevant applications. Overall, we hope that this forward-looking perspective will initiate the integration of Raman spectroscopy into the field of ecology and evolution.
